# Mechanistic Insights into Angiotensin I-Converting Enzyme Inhibitory Tripeptides to Decipher the Chemical Basis of Their Activity

**DOI:** 10.1021/acs.jafc.2c04755

**Published:** 2022-09-08

**Authors:** Carmen Lammi, Giovanna Boschin, Martina Bartolomei, Anna Arnoldi, Gianni Galaverna, Luca Dellafiora

**Affiliations:** † Department of Pharmaceutical Sciences, University of Milan, Via Mangiagalli 25, Milan 20133, Italy; ‡ Department of Food and Drug, University of Parma, Parco Area delle Scienze 27/A, Parma 43124, Italy

**Keywords:** structure−activity relationship, bioactive peptides, angiotensin I-converting enzyme, antihypertensive peptides

## Abstract

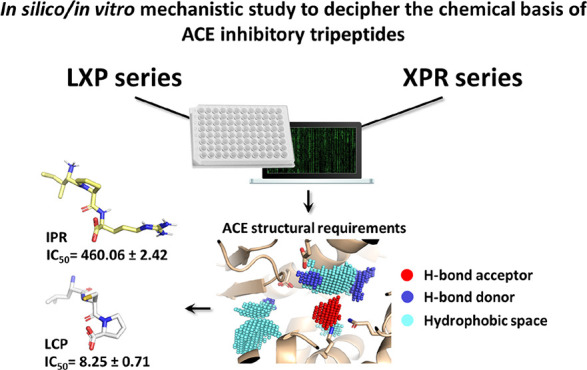

Food proteins are an important source of bioactive peptides, and the angiotensin I-converting enzyme (ACE) inhibitors are worthy of attention for their possible beneficial effects in subjects with mild hypertension. However, the chemical basis underpinning their activity is not well-understood, hampering the discovery of novel inhibitory sequences from the plethora of peptides encrypted in food proteins. This work combined computational and *in vitro* investigations to describe precisely the chemical basis of potent inhibitory tripeptides. A substantial set of previously uncharacterized tripeptides have been investigated *in silico* and *in vitro*, and LCP was described for the first time as a potent ACE inhibitory peptide with IC_50_ values of 8.25 and 6.95 μM in cell-free and cell-based assays, respectively. The outcomes presented could serve to better understand the chemical basis of already characterized potent inhibitory tripeptides or as a blueprint to design novel and potent inhibitory peptides and peptide-like molecules.

## Introduction

Food proteins have important roles beyond the well-characterized nutritional properties, and their importance as a source of health-promoting bioactive peptides has been intensively investigated since decades.^1^ Bioactive peptides are referred to as amino acid sequences, generally 3–20 residues in length, encrypted in food proteins that can exert many biological activities after they are released during digestion and absorbed through gastrointestinal epithelium.^2^ The regulation of blood pressure, the reduction of the cholesterol level, and the effects on glucose metabolism are among the most studied effects of bioactive peptides to cite but a few.^3^ In particular, the capacity of certain bioactive peptides to inhibit the angiotensin I-converting enzyme (ACE; EC 3.4.15.1) has been causatively linked to a beneficial reduction of blood pressure in living organisms.^4,5^ ACE is a carboxy-dipeptidase with two catalytically active domains (namely, N- and C-terminal domains) converting the inactive peptide angiotensin I into the potent vasoconstrictor hormone angiotensin II and having a key role in regulating blood pressure.^6^ Its inhibition may result in an effective reduction of blood pressure in living organisms with beneficial effects in subjects suffering from hypertension.^7^

Protein hydrolysates that contain bioactive peptides could be used to develop functional foods and bioactive peptides *per se* to produce nutraceuticals for nonpharmacological applications.^8^ However, the use and design of these products requires a deep understanding of the chemical and molecular basis of peptide bioactivity to grasp with precision fit-for-purpose bioactive sequences from the plethora of peptides encrypted in food proteins. Currently, such understanding is still far from being complete. Nonetheless, the current state-of-the-art has showed that short peptides (dipeptides and tripeptides) are prevalent among the most potent ACE inhibitory peptides of food origin described so far.^9^ Of note, the capability of bioactive sequences to resist hydrolysis during gastrointestinal digestion, absorption, and distribution through the body is crucial to ensure effects on living organisms. In this respect, tripeptides deserve a particular attention in the light of their relatively high bioavailability, potency, gastric stability, and absorption by enterocytes.^10^ Tripeptides have been widely investigated in the past, providing useful—although partial—insights into the structural requirements underpinning their capacity to inhibit ACE. Specifically, a C-terminal proline (third position) generally enhances the inhibitory activity while positively charged and hydrophobic amino acids are preferred at the second and first (N-terminal) position, respectively. In line with this evidence, the LXP series includes potent *in vitro* ACE inhibitory peptides (as per the BIOPEP-UWM database; https://biochemia.uwm.edu.pl/en/biopep-uwm-2).^11^ As an example, LKP and LMP are among the most potent *in vitro* ACE inhibitory tripeptides identified so far, with *in vivo* effects being reported for the former and expected for the latter.^4,12^ Nevertheless, the mechanistic basis of such a structure–activity relationship, including but not limited to the clear description of how inhibitory tripeptides chemically interact with ACE and why the enzyme either refuses or prefers to being inhibited by certain sequences, still needs clarifications. This missing piece of information ultimately hampers an informed and precise identification/design of potent ACE inhibitory tripeptides.

In this context, this work combined *in silico* analysis and *in vitro* trials presenting a rational, useful, and straightforward strategy either to better understand the 3D structure–activity relationship of inhibitory tripeptides or to identify new, potent, and previously uncharacterized ACE inhibitory sequences. Briefly, members of the LXP and XPR series never characterized before were studied *in silico* and *in vitro* to identify strong candidates for further and more detailed investigations. Among the active sequences, LCP has been identified for the first time to the best of our knowledge as a novel and potent *in vitro* ACE inhibitory peptide. In addition, the relevance of our outcomes to design or identify new ACE inhibitory molecules and broaden the horizon of knowledge of ACE inhibitory tripeptides was discussed.

## Materials and Methods

### Computational Analysis

#### Construction of the Tripeptide Library

The 3D structures of tripeptides analyzed in this study were automatically generated in the Trypos “.mol2” format using the Biopolymer tool implemented in Sybyl, version 8.1 (https://www.certara.com) through an in-house Sybyl programming language script as described previously.^12^ In more detail, peptides were designed using the “Build Protein” tool setting the N- and C-terminus as protonated and deprotonated, respectively. Prior to subsequent analysis, each peptide was energetically minimized using the Powell algorithm with a coverage gradient of ≤0.05 kcal mol^–1^ Å^–1^ and a maximum of 500 cycles, in agreement with a previous study.^14^

#### Docking Analysis

The capability to inhibit ACE was estimated for a selection of sequences through docking simulations using GOLD software (genetic optimization for ligand docking, version 2021.10) in agreement with previous studies.^12,15,16^ In brief, the binding poses were scored using the GOLDScore scoring function, which considers the external (protein–peptide complex) and internal (peptide only) van der Waals energy, protein–peptide hydrogen bond energy, and ligand torsional strain energy, and only the best-scored pose for each peptide has been considered for the analysis (the higher the score, the better the theoretical fitting within the pocket). Furthermore, a semi-flexible docking approach was applied while allowing ACE polar hydrogens to rotate freely and considering peptides fully flexible. The models for both C- and N-domains of ACE were derived from the Protein Data Bank (http://www.rcsb.org) structures having PDB codes 4APH and 4BZS, respectively,^17^ as previously described.^15^ In addition, for each peptide, simulations were run in triplicate (scores are expressed as means ± standard deviations) as GOLD adopts a genetic algorithm that may cause score fluctuations. The scores were found to be satisfactorily stable with a maximum coefficient of variation of 7% for YPR and less than 5% for all the other sequences, in agreement with previous studies.^18^

#### Pharmacophoric Analysis

The anatomy of ACE’s pockets was defined with GetCleft,^19^ while the respective pharmacophoric fingerprints were derived using the IsoMIF algorithm,^20^ as previously described.^12^

#### Molecular Dynamics Simulations

Molecular dynamics simulations were used to estimate the stability of ACE in complex with LCP, in agreement with a previous study,^21^ using GROMACS (version 2019.4),^22^ solvating input structures with SPC/E waters under a dodecahedron periodic boundary condition and neutralizing the system with counterions (Na^+^ or Cl^–^). In brief, each system underwent an energy minimization to avoid steric clashes and to correct improper geometries using the steepest descent algorithm with a maximum of 5,000 steps. Then, all the systems underwent isothermal (300 K, coupling time 2 psec) and isobaric (1 bar, coupling time 2 psec) 100 psec simulations before running 50 nsec simulations (300 K with a coupling time of 0.1 psec and 1 bar with a coupling time of 2.0 psec).

#### Statistical Analysis of Docking Results

The docking simulations were run in triplicates, and the statistical analysis of docking results was performed using IBM SPSS Statistics for Linux, version 25 (IBM Corp., Armonk, NY).

### Experimental Analysis

#### Chemicals and Sampling

All chemicals (reagents and solvents) were from Sigma-Aldrich (St. Louis, MO, USA). Caco-2 cells were obtained from INSERM Paris, France; Dulbecco’s modified Eagle’s medium (DMEM), stable l-glutamine, foetal bovine serum (FBS), phosphate-buffered saline, penicillin/streptomycin, and 96-well plates were purchased from Euroclone (Milan, Italy). The ACE1 activity assay kit was from Biovision (Milpitias, CA, USA). The peptides LKP and LCP were synthetized by GenScript (Piscataway, NJ, USA) at >95% purity.

#### *In Vitro* Evaluation of ACE Inhibitory Activity

Peptides were tested as already described^23^ evaluating hippuric acid (HA) formation from hippuryl–histidyl–leucine (HHL), as a mimic substrate for angiotensin I.

#### Cell Line Culture

Caco-2 cells were routinely sub-cultured at 50% density and maintained at 37 °C in a 90% air/10% CO_2_ atmosphere in DMEM containing 25 mM glucose, 3.7 g/L NaHCO_3_, 4 mM stable l-glutamine, 1% non-essential amino acids, 100 U/L penicillin, and 100 μg/L streptomycin (complete medium), supplemented with 10% heat-inactivated FBS (Hyclone Laboratories, Logan, UT, USA).

#### 3-(4,5-Dimethylthiazol-2-yl)-2,5-diphenyltetrazolium Bromide (MTT) Assay

A total of 3 × 10^4^ Caco-2 cells/well were seeded in 96-well plates and treated with 0.1–100 μM LCP or vehicle (H_2_O) in complete growth media for 48 h at 37 °C under a 5% CO_2_ atmosphere. Subsequently, the treatment solvent was aspirated, and 100 μL/well of 3-(4,5-dimethylthiazol-2-yl)-2,5-diphenyltetrazolium bromide (MTT) filtered solution was added. After 2 h of incubation at 37 °C under a 5% CO_2_ atmosphere, 0.5 mg/mL solution was aspirated and 100 μL/well of the lysis buffer (8 mM HCl + 0.5% NP-40 in DMSO) was added. After 10 min of slow shaking, the absorbance at 575 nm was read on a Synergy H1 fluorescence plate reader (Biotek, Bad Friedrichshall, Germany).

#### Cell-Based ACE Activity Assay

Caco-2 cells (5 × 10^4^ cells/well) were seeded in 96-well plates. After 24 h, cells were treated with 100 μL of LCP peptides (from 1.0 to 50.0 μM) or vehicle in growth medium for 24 h at 37 °C. The next day, cells were scraped in 30 μL of ice-cold ACE1 lysis buffer and centrifuged at 13,300 g for 15 min at 4 °C. Total proteins were quantified from the supernatant by the Bradford method, and 1.5 μg of total proteins (the equivalent of 1.5 μL) was added to 18 μL of ACE1 lysis buffer in each well in a black 96-well plate with clear bottoms. For the background control, 20 μL of ACE1 lysis buffer was added to 20 μL of ACE1 assay buffer. Then, 20 μL of 4% ACE1 substrate (in assay buffer) was added in each well except the background one, and the fluorescence (Ex/Em 330/430 nm) was measured in a kinetic mode for 10 min at 37 °C.

#### Statistical Analysis of Biological Experiments

*In vitro* (cell-free) and cellular ACE activity for each peptide was expressed as a mean value of four independent experiments in triplicate ± standard deviation. All the data sets were checked for normal distribution by D’Agostino and Pearson test. Since they were all normally disturbed with *p*-values < 0.05, the existence of significant differences in terms of activity for AKP, LCP, and LKP in cell-free or cell-based tests was calculated by Student’s t-test using Graph-pad Prism 9 (SanDiego, CA, USA; *p*-values < 0.05).

## Results and Discussion

Computational methodologies already proved to be effective means to study bioactive peptides of food origin.^24^ Particularly, the 3D molecular modeling pipeline used here, which consisted in docking studies, pharmacophoric analysis, and molecular dynamic simulations, already proved to be a reliable means to predict the activity of ACE inhibitory peptides.^12,15,21^ Therefore, it was used to assess the inhibitory potential of uncharacterized members of the LXP series prior to experimental trials. In this respect, the LXP series includes some of the most potent ACE-inhibitory peptides identified so far, like LKP, LMP, and LGP to cite but a few (as per BIOPEP-UWM database; https://biochemia.uwm.edu.pl),^11^ with an *in vitro* reported activity (IC_50_) ranging from 0.32 μM (LKP) to 57 μM (LLP) (Table S1; Supporting Information). However, the gold benchmark database for bioactive peptides BIOPEP-UWM reported no data for 9 out of 20 series members (*i.e.*, LEP, LDP, LIP, LTP, LCP, LHP, LFP, LWP, and LVP; last database access 7th December 2021), which were analyzed here using a knowledge-based approach as follows. Specifically, the current understanding of the structure–activity relationship of ACE-inhibiting tripeptides clearly suggests an acid residue at the second position is likely to reduce the activity and led to exclude LEP and LDP from further analysis.^9,12,25^ LIP and LTP were also excluded since they were analyzed in a previous work with the same pipeline described here, showing a theoretical inhibitory activity worse than LKP.^12^ In addition, LIP sequence was highly similar to LLP, the least potent member of the LXP series with an IC_50_ of 57 μM (Table S1), and it was not expected to have a strong inhibitory activity accordingly. The remaining uncharacterized sequences LCP, LHP, LFP, LWP, and LVP were selected for the analysis in order to identify promising candidates for further experimental investigations. AKP, which is a close analogue of LKP, was also analyzed to further test the hypothesized importance of bulky hydrophobic amino acid first position (see above).

**Figure 1:**
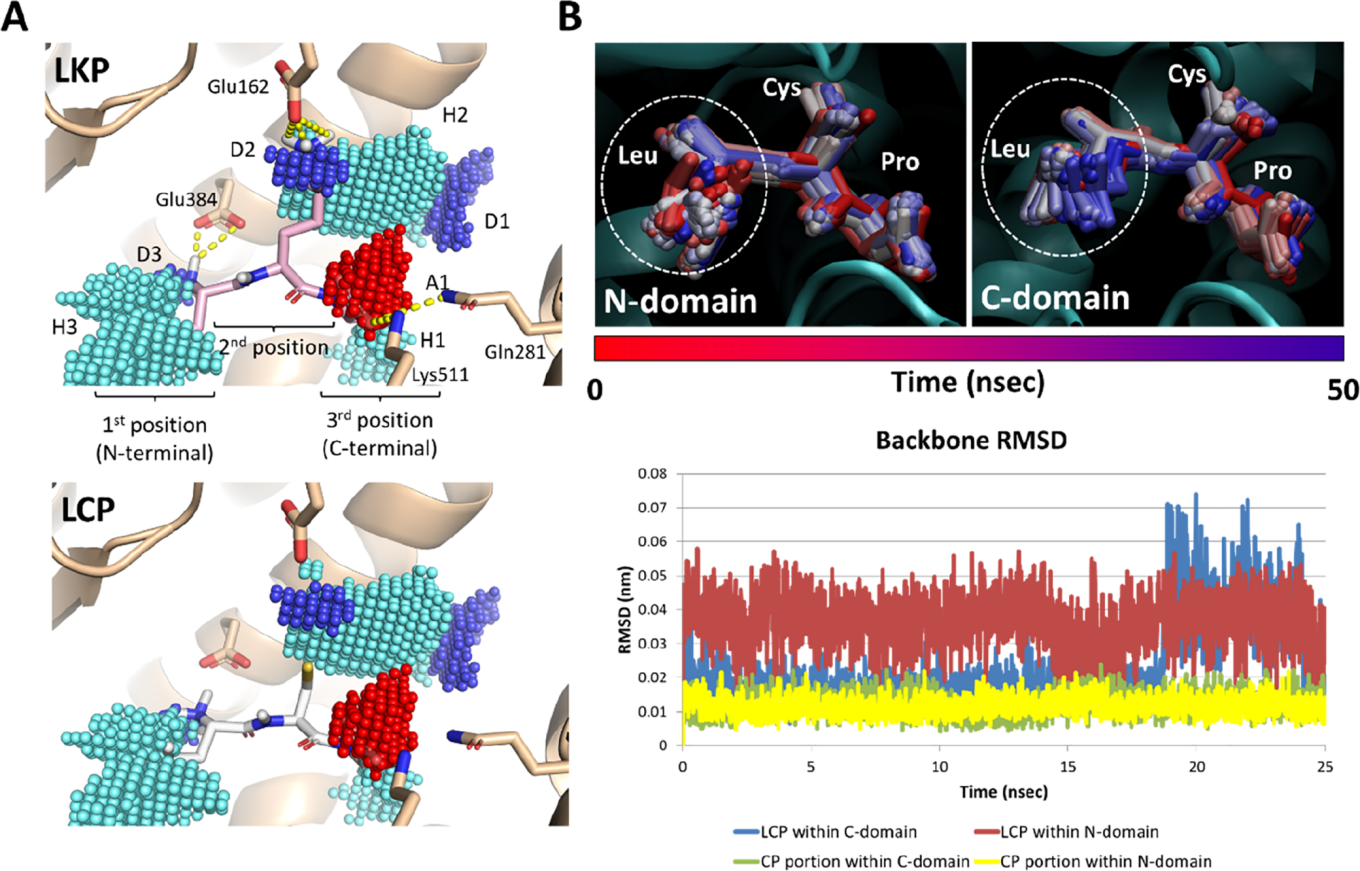
Computational results of LKP and LCP. (A) Pharmacophoric analysis of ACE C-domain binding site and docking poses of LKP and LCP. Protein is represented in cartoon and peptides in sticks. Yellow dashed lines indicate polar contacts. Cyan, red, and blue spheres indicate areas able to receive hydrophobic, hydrogen bond acceptor, or hydrogen bond donor groups, respectively. (B) Results of molecular dynamics. The top figure reports the time step representation of LCP trajectories (represented in sticks) within the N- and C-terminal domain of the ACE (represented in cartoon). The red-to-blue color switch indicates the stepwise changes of peptide coordinates along the simulation. The white dashed ring indicates the position of Leu at the first position (N-terminal). The figure in the bottom reports the RMSD analysis of LCP.

These sequences underwent docking studies first, and the respective docking poses were analyzed in the light of the current understanding of the structure–activity relationship and the pharmacophoric fingerprint of ACE pockets. As shown in Table 1, all of them recorded relatively high scores in line with those of LKP, taken as the reference compound, pointing to their theoretical capacity to favorably arrange into the ACE binding pockets (the score is proportional to the theoretical capability of ligands to fit the protein pocket). Docking poses were then analyzed in the light of the pharmacophoric fingerprint of ACE pockets. The small differences among the two ACE domains were found not relevant for the sake of this study, and therefore, only the results concerning the C-terminal domain are presented for simplicity, in agreement with previous studies.^12,15^ All the sequences analyzed showed a comparable architecture of binding with both the C- and N-terminal engaged in polar contacts with the enzyme, in agreement with previous studies.^12^ The close inspection of the pharmacophoric fingerprint of the ACE substrate-binding site revealed a well-defined distribution of areas able to receive hydrophobic groups or polar groups like hydrogen bond donors and acceptors. In more detail, a pharmacophoric map with six different districts was defined, and such a map could explain the activity of peptides belonging to the LXP series characterized so far (Figure 1). Three main hydrophobic areas, namely, H1, H2, and H3, were found to be able to receive the hydrophobic portion of the side chain at the third (C-terminal), second, and first (N-terminal) position, respectively. In addition, two out of three regions able to receive hydrogen bond donors were found to accept the peptide’s amino terminal and the positively charged (ε-) amino group at the second position (D2 and D3, respectively). Finally, an area suitable for receiving hydrogen bond acceptors was found occupied by the peptide’s carboxylic group at the carboxy terminal (A1). A heuristic interpretation of the data collected so far for LXP series members (Table S1) in the light of the pharmacophoric map described here suggested that side chains with polar (hydrogen bond donors) groups are strongly preferred at the second position when they satisfy the hydrophobic requirements of the pocket and, at the same time, can engage Glu162 with polar contacts (as per LKP and reasonably for LRP). Such an interaction can contribute to the ACE–peptide binding with hydrophobic–hydrophobic favorable contributions arranging the hydrophobic neck of the side chain within H2 and with polar interactions due to the positively charged terminus of the side chain being arranged within D2. Conversely, the presence of uniquely hydrophobic side chains seemed less preferred at this position as they could satisfy only the H2 area, reducing thereby the overall number of favorable contributions to the binding event and resulting in a lower inhibitory capacity. Also, the activity seemed inversely linked to the dimension of the hydrophobic side chain. Indeed, LAP and LGP had relatively low IC_50_ while peptides with a bulkier hydrophobic side chain like LLP and LMP recorded a slightly bad activity compared to them (as per the BIOPEP-DW database, Table S1). On this basis and in the light of the docking poses calculated for the set of peptides analyzed here, LCP was identified as the most promising peptide to test with experimental trials among the LXP members considered here. Indeed, LHP, LFP, LWP, and LVP were not able to engage Glu162, and they were found arranging the side chain at the second position within the hydrophobic H2 area (Figure S1; Supporting Information). On this basis, they were considered similar to LLP or LMP, and an activity lower than LKP was expected for them accordingly although they could not be ranked quantitatively based on docking scores (which were relatively high) and the similar fitting they showed within the pharmacophoric space of the pocket (Figure S1; Supporting Information). Conversely, LCP showed Cys at the second position that had a small and hydrophobic side chain^26^ and could allow a relatively potent inhibition, possibly lower than that of LKP and similar to that of LAP and LGP (Table S1; Supporting Information). The capability of LCP to interact with ACE was also evaluated with molecular dynamics simulations to assess the geometrical stability of such interaction, in agreement with previous studies.^12,21^ As shown in Figure 1B, the trajectory and root-mean-squared deviation (RMSD) analysis of LCP revealed its capacity to stably interact with both ACE domains in the timeframe considered. In more details, in both domains, the Leu at the first position (N-terminal) was found more mobile compared to the other two residues and was also responsible for the slight increase in RMSD values observed from 18 to 23 nanoseconds in complex with the C domain. Conversely, the second and third residues were found very stable with a steady-state RMSD trend during the whole simulation. On this basis, the interaction of LCP with ACE was predicted to be stable, and a high inhibitory capacity was expected accordingly. In addition, to further study the structure–activity relationship of LXP series, AKP was also calculated to further investigate the role of bulky hydrophobic residues at the first position. Docking analysis showed a geometry of binding very similar to that of LKP. However, a lower hydrophobic–hydrophobic favorable contribution to the binding event was inferred as the side chain of Ala was less embedded into the hydrophobic contour H3 (Figure S2; Supporting Information) compared to the Leu’s side chain of LKP. A lower interaction and a worse activity compared to LKP was hypothesized for AKP accordingly.

**Table 1:** Docking and Experimental Results of the *In Vitro* ACE Inhibitory Activity

		docking scores^a^		experimental assessment	
sequence		N-domain	C-domain	ACE-inhibition (%)^b^	IC_50_ (μM)^c^
AKP		69.47 ± 1.04	88.29 ± 4.47	86.14 ± 0.35	719.90 ± 16.04
LXP series	LKP	76.15 ± 0.97^d^	95.20 ± 3.31^d^	97.36 ± 0.15	9.23 ± 0.56^d^
	LCP	69.70 ± 0.82	85.93 ± 2.45	98.38 ± 0.31	8.25 ± 0.71
	LHP	71.74 ± 0.70	87.97 ± 2.14	n.d.	n.d.
	LFP	72.97 ± 1.56	90.07 ± 0.76	n.d.	n.d.
	LWP	78.73 ± 1.34	90.55 ± 0.59	n.d.	n.d.
	LVP	71.42 ± 1.22	74.34 ± 1.09	n.d.	n.d.
XPR series	YPR	85.58 ± 3.80	92.52 ± 6.72	46.64 ± 0.62	n.d.
	GPR	83.02 ± 2.67	85.44 ± 3.33	16.36 ± 1.46	n.d.
	IPR	88.79 ± 1.64	89.74 ± 2.04	84.58 ± 0.46	460.06 ± 2.42
	NPR	86.31 ± 2.84	79.26 ± 1.43	44.60 ± 0.42	n.d.

a Docking scores are expressed as mean values ± standard deviation of three independent docking simulations.

b ACE-inhibition percentage obtained with AKP at 3.2 mM, LKP and LCP at 0.3 mM, whereas with YPR, GPR, IPR, and NPR at 2.3, 3, 2.6 and 2.6 mM, respectively, expressed as a mean value of four independent experiments in triplicate ± standard deviation.

c IC_50_ stands for the half maximal inhibitory concentration, and it is expressed as a mean value of four independent experiments in triplicate ± standard deviation unless otherwise indicated.

d According to^12^; n.d. stands for “not determined” in this work.

LCP and AKP were then selected for *in vitro* analysis based on computational outcomes to characterize experimentally a strong ACE inhibitory candidate peptide and to further test the role of hydrophobic side chains at the first position of LXP series, respectively. The *in vitro* (cell-free) ACE inhibitory potential was evaluated using the porcine kidney recombinant enzyme taking the results of LKP previously described^12^ as reference. LCP and AKP efficiently inhibited the ACE activity by 98.38 ± 0.31% and 86.14 ± 0.35% at 0.3 and 3.2 mM, respectively. The high inhibitory activity found under cell-free conditions supported their assessment in cell-based tests. LCP displays an IC_50_ value equal to 8.25 ± 0.71 μM, whereas AKP displays an IC_50_ value equal to 719.90 ± 16.04 μM (Table 1). For the first time, LCP was described as a potent inhibitory peptide equivalent to LKP (no significant differences were observed by statistical analysis), while AKP showed a very weak inhibitory activity (*p* < 0.05). These results agreed with the *in silico* outcomes and confirmed the importance of a bulky hydrophobic residue at the first position (N terminal) and small hydrophobic side chains at the second position. Furthermore, LCP was selected for further characterization in cell-based assay due to the high activity showed under the cell-free conditions. In this respect, the existence of cytotoxic effects in the test system used, which was based in the human intestinal Caco-2 cells, was excluded *via* MTT experiments, observing no effects on the cellular vitality in the range of concentration 0.1–100 μM (Figure S3). On this basis, Caco-2 cells were treated with LCP and LKP, taken as reference compounds, in the range of concentration 0.1–100 μM for 24 h to evaluate the effects on the ACE activity expressed at the human cellular level. Cells then underwent lysis, and the ACE activity was measured in the presence of a fluorescent substrate (see Materials and Methods for further details). LCP and LKP effectively reduced the cellular ACE activity with a dose–response curve and an IC_50_ value equals to 6.95 ± 0.29 and 3.71 ± 0.23 μM (Figure 3).

**Figure 2:**
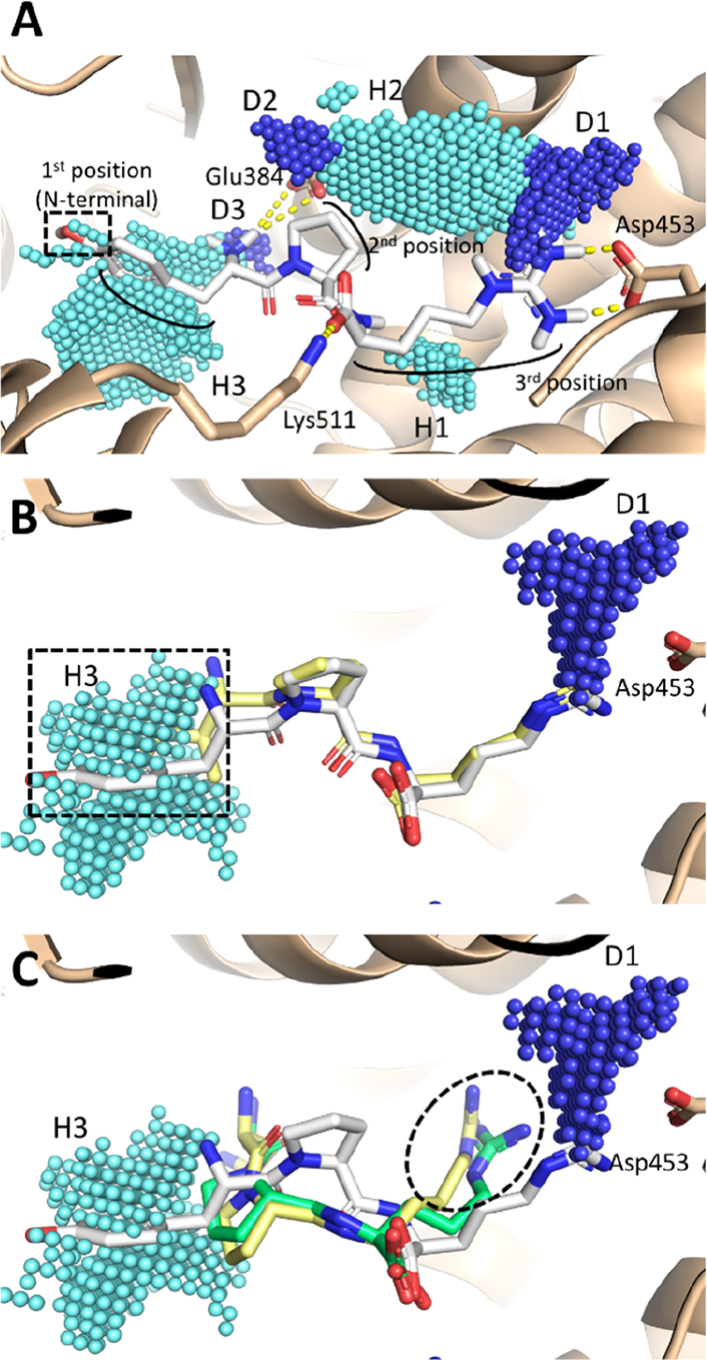
Docking results of YPR, GPR, IPR, and NPR. Protein is represented in cartoon and peptides in sticks. Cyan and blue spheres indicate areas able to receive hydrophobic or hydrogen bond donor groups, respectively. (A) Binding pose of YPR. Polar contacts are represented by yellow dashed lines while the black dashed box indicates the improper arrangement of hydroxyl groups into a hydrophobic space. (B) Binding pose of IPR (yellow-colored) compared to that of YPR (white-colored). The black dashed box indicates the arrangement of the side chain at the first position (N-terminal). (C) Binding pose of GPR (yellow-colored) and NPR (green-colored) compared to that of YPR (white-colored). The black dashed circle indicates the different arrangement of the Arg side chain at the third position (C-terminal) of GPR and NPR compared to YPR.

**Figure 3:**
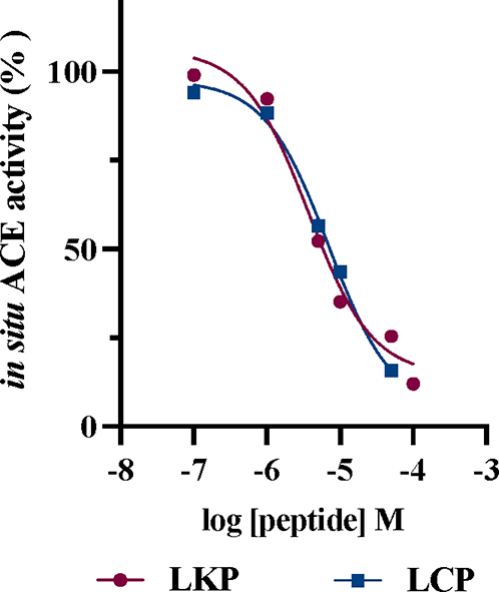
Dose-response effect of the LCP peptide on the ACE activity expressed by human intestinal Caco-2 cells.

Notably, to the best of our knowledge, LCP was described for the first time as a potent ACE inhibitory peptide with an IC_50_ value similar to that of LKP and in the range of activity of the most potent ACE inhibitory peptides identified so far (Table 1). Importantly, previous evidence proved the efficacy of LKP to reduce the blood pressure spontaneously in hypertensive rats after oral administration.^4^ Therefore, the structural analogies might suggest a certain degree of activity *in vivo* also for LCP that may deserve further dedicated investigations. The identification of relevant food sources and the actual release of LCP during digestion, considering that Cys may be involved in disulfide bonds possibly impairing protein hydrolysis and peptides release, are among those with the highest priority to finally assess the relevance of LCP from a real-world perspective.

The pharmacophoric analysis drew also the attention on a region suitable to receive hydrogen bond donors (D1; Figure 1) close to the hydrophobic region receiving the side chain of residues in 2nd position (H2; Figure 1). The D1 region is unoccupied by members of LXP series, but it was hypothesized likely to receive the polar side chain of Arg or Lys providing ground to better understand the mechanistic basis of inhibitory peptides with Arg or Lys at the first position (C-terminal). The analysis of tripeptides reported in the benchmark database BIOPEP-UWM revealed the presence of YPR among the most active peptides with a reported IC_50_ of 16.5 μM (https://biochemia.uwm.edu.pl). The presence of Pro, which is a small and hydrophobic residue, at the second position agreed with the pocket requirements described above. Tyr at the first position (N terminal) was not only likely to fit the pharmacophoric space of the pocket in the H3 area but also supposed to cause a certain degree of hydrophobic/polar interferences due to the possible arrangement of its phenolic portion within the hydrophobic area H3. To verify this hypothesis, YPR underwent docking analysis, which revealed the same binding architecture of LXP series, the extension of the Arg side chain into the D1 area (engaging Asp453 with polar contacts), and the actual arrangement of the Tyr phenolic group into the hydrophobic area H3, possibly causing hydrophobic/polar interferences (Figure 2A). YPR was investigated *in vitro* showing an ACE inhibitory percentage of nearly 50% at 2.3 mM (Table 1) and was not assessed with cell-based assays due to the relatively low activity observed. Nonetheless, a selection of XPR series members, that is, GPR, IPR, and NPR, was analyzed *in silico* and *in vitro* to extend the understanding of the structure–activity relationship for the XPR series. Such sequences were chosen to test the effect of the side chain at the first position (N-terminal) taking IPR and NPR as representatives for bulky hydrophobic and polar side chains, respectively, and GPR to observe the effects when the side chain is missing. As shown in Table 1, their activity ranged from mild to weak, with IPR being described as the most active with an *in vitro* (cell-free) ACE inhibition of nearly 85% and IC_50_ of 460.06 ± 2.42 μM. None of the sequences belonging to the XPR series other than IPR were considered for the further experiments in cells due to the low activity observed in cell-free conditions (Table 1). However, computational analysis could explain the experimental evidence from a mechanistic standpoint. As shown in Table 1, GPR, IPR, and NPR were all found to be able to satisfy pocket requirements as they all recorded relatively high docking scores. However, the analysis of docking poses revealed substantial differences in the mode of binding. Although they all showed a comparable architecture of binding, YPR and IPR, but not GPR and NPR, arranged the guanidinium group of Arg at the third position (C-terminal) within the D1 area engaging with polar contacts Asp 453. This missing interaction could reasonably result in a weak interaction with the ACE for GPR and NPR, which may have caused their limited inhibitory capacity compared to that of YPR and IPR. In addition, IPR showed the hydrophobic side chain at the first position (N-terminal) well-embedded into the hydrophobic area H3, and the lack of hydrophobic/polar interference described for YPR could explain the higher activity of the former.

Taken together, these results highlighted the structural basis underpinning the importance of bulky hydrophobic residues at the first position (N-terminal) of ACE inhibitory tripeptides. We also detailed why the second position should have either small hydrophobic amino acids or bulky side chains with a hydrophobic neck and a polar head for an optimal engagement of the surrounding environment of the pocket. We also showed that further polar contacts *via* the side chain at the first position (C-terminal) seemed not effective to substantially enhance the inhibitory potential of tripeptides.

In conclusion, this study applied a 3D molecular modeling approach to understand the structural requirements of a potent ACE inhibitory tripeptide from a mechanistic point of view, providing a detailed pharmacophoric map able to explain their activity with precision. Of note, understanding the chemical rationale of peptides bioactivity may either lead to make predictions on previously untested sequences, as per LCP, or to extend the mechanistic understanding of peptides that previously tested active. Computational predictions were confirmed *in vitro*, and LCP was described as a potent ACE inhibitory peptide for the first time to the best of our knowledge. The sequence and mechanistic analogies with LKP, a sequence previously proved active *in vivo*, ultimately pointed out the relevance of further dedicated investigations. Generally speaking, the methodology presented here showed to be an effective and reliable method to investigate the ACE inhibitory activity of peptides and provided a characterization of ACE inhibitory activity for a substantial set of sequences. Moreover, the structural outcome presented here could serve either as a foothold to better understand the chemical basis underpinning the activity of already characterized inhibitory tripeptides or as a blueprint to design novel and potent inhibitory peptides and peptidomimetic molecules. In addition, the geometries of binding presented may allow to design site-specific mutagenesis and crystallographic studies to experimentally confirm the key contacts between tripeptides and ACE as a key piece of information to design novel peptido-mimetic inhibitors. In this respect, all the 3D-coordinate files of peptides and peptide–protein complexes are available upon request.

## Supporting Information Available

List of sequences from LXP and XRP series and respective experimental activity reported in the literature; pharmacophoric analysis of ACE C-domain binding sites and docking poses of LKP, LFP, LHP, LVP, and LWP; docking pose of AKP and LKP; and experimental data of LCP’s effects on the Caco-2 cells’ viability (PDF)

## Abbreviations

ACE: angiotensin I-converting enzyme
DMEM: Dulbecco’s modified Eagle’s medium
FBS: foetal bovine serum
GOLD: genetic optimization for ligand docking
HP: hippuric acid
MTT: 3-(4,5-dimethylthiazol-2-yl)-2,5-diphenyltetrazolium bromide
PBS: phosphate-buffered saline
PDB: protein data bank
RMSD: root-mean-squared deviation
