# Supplementary material for: Mechanistic Insights into Angiotensin I-Converting Enzyme Inhibitory Tripeptides to Decipher the Chemical Basis of Their Activity
Source: J Agric Food Chem. 2022 Sep 8;70(37):11572–8. doi: 10.1021/acs.jafc.2c04755 (PMC9501895; doi:10.1021/acs.jafc.2c04755)
Supplement: Supplementary file 1 — jf2c04755_si_001.pdf [file jf2c04755_si_001.pdf]

Supporting information

Mechanistic insights on angiotensin I converting enzyme inhibitory peptides to decipher the chemical basis of their activity

Carmen Lammi [1], Giovanna Boschin [1], Martina Bartolomei [1], Anna Arnoldi [1], Gianni Galaverna [2], Luca Dellafiora [2*]

[1] Department of Pharmaceutical Sciences, University of Milan, Via Mangiagalli 25, 20133 Milan, Italy.

[2] Department of Food and Drug, University of Parma, Parco Area delle Scienze 27/A, 43124 Parma, Italy.

* Correspondence to: Luca Dellafiora, Department of Food and Drug, University of Parma, Parco Area delle Scienze 27/A, 43124 Parma, Italy. Email: luca.dellafiora@unipr.it; Phone: +39 0521 906079

Table S1. List of sequences from LXP and XRP series and respective activity*

Sequence	Activity (IC_{50}; µM)	Sequence	Activity (IC_{50}; µM)
LNP	43	YPR	16.5
LYP	6.6	NPR	285.4
LGP	0.72	LPR	Unknown
LSP	1.7	GPR	Unknown
LRP	1	SPR	Unknown
LKP	0.32	RPR	Unknown
LQP	1.9	KPR	Unknown
LAP	3.5	QPR	Unknown
LMP	15.8	APR	Unknown
LPP	9.6	MPR	Unknown
LLP	57	PPR	Unknown
LEP	Unknown	EPR	Unknown
LDP	Unknown	DPR	Unknown
LIP	Unknown	IPR	Unknown
LTP	Unknown	TPR	Unknown
LCP	Unknown	CPR	Unknown
LHP	Unknown	HPR	Unknown
LFP	Unknown	FPR	Unknown
LWP	Unknown	WPR	Unknown
LVP	Unknown	VPR	Unknown

* Experimental activity, expressed as IC_{50} (half maximal inhibitory concentration), reported in the BIOPEP-UWM database (last database access 21st December 2021; http://www.uwm.edu.pl/biochemia/index.php/en/biopep)

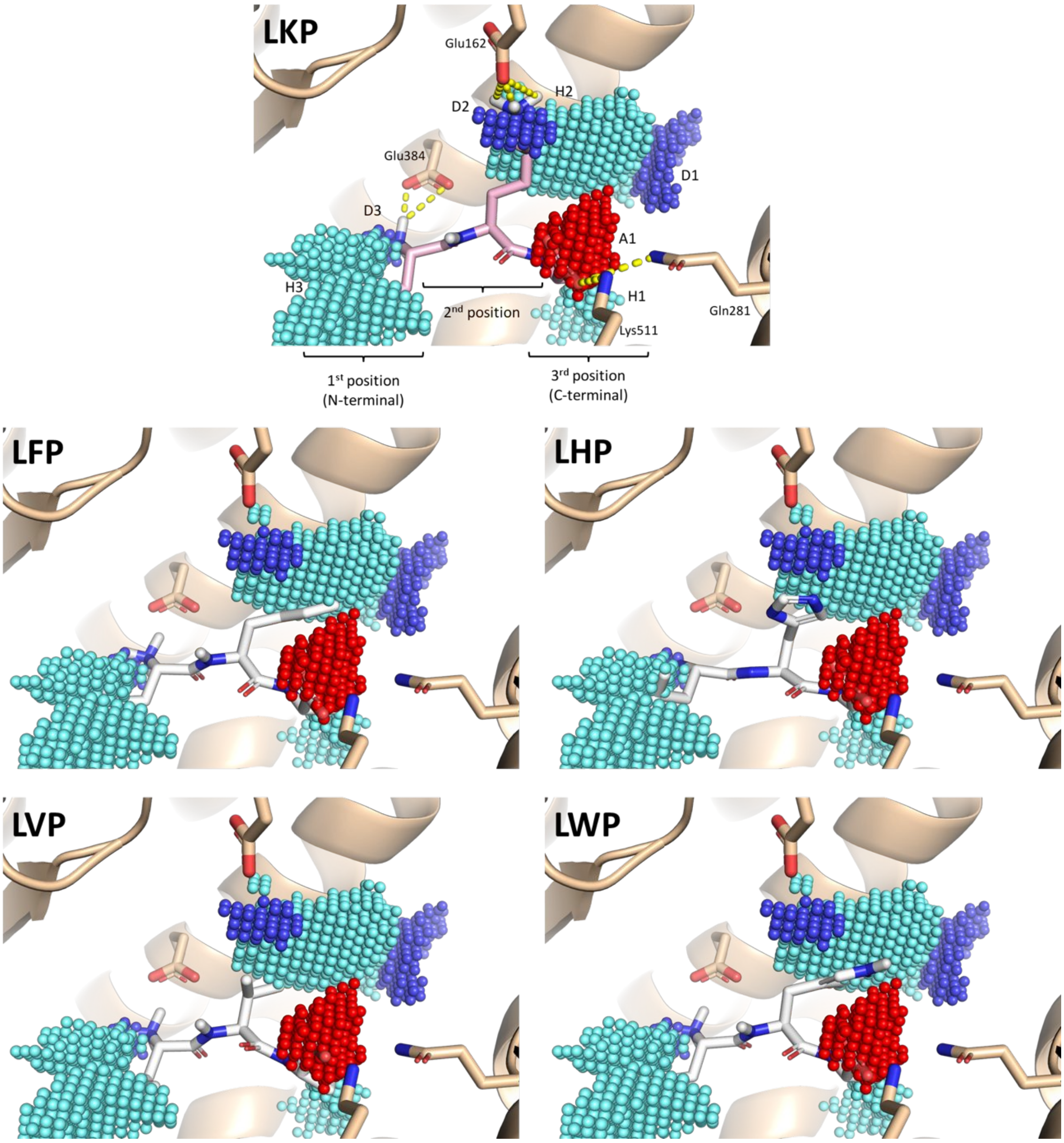

Figure 1S. Pharmacophoric analysis of ACE C-domain binding site and docking poses of LKP, LFP, LHP, LVP and LWP. Polar contacts are represented by yellow dashed lines. Cyan, red and blue spheres indicate areas able to receive hydrophobic, hydrogen-bond acceptor or hydrogen-bond donor groups, respectively.

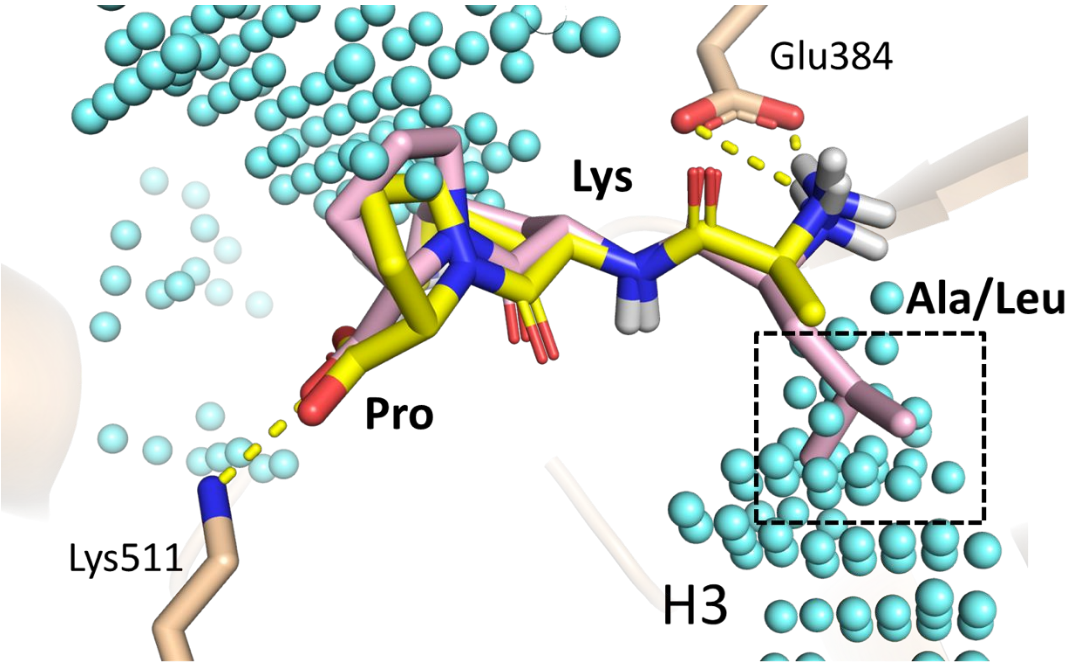

Figure 2S. Docking pose of AKP (coloured in yellow) and LKP (coloured in white). Polar contacts are represented by yellow dashed lines. Cyan spheres indicate areas able to receive hydrophobic groups. Tha black dashed box indicates side chain of Leu at the N-terminal of LKP well embedded into the hydrophobic contour, while such favourable hydrophobic/hydrophobic interaction is missing for AKP.

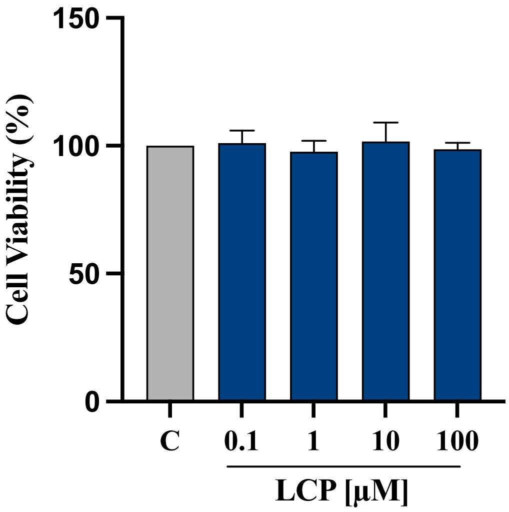

Figure 3S. Effect of LCP on the Caco-2 cells' viability
